# Epigenetics in age-related macular degeneration: new discoveries and future perspectives

**DOI:** 10.1007/s00018-019-03421-w

**Published:** 2020-01-02

**Authors:** M. Gemenetzi, A. J. Lotery

**Affiliations:** 1 NIHR Biomedical Research Centre At Moorfields Eye Hospital NHS Foundation Trust and UCL Institute of Ophthalmology, 162 City Road, London, EC1V 2PD UK; 2 Clinical and Experimental Sciences, Faculty of Medicine, University Hospital Southampton, University of Southampton, South Lab and Path Block, Mailpoint 806, Level D, Southampton, SO16 6YD UK

**Keywords:** Age-related macular degeneration, Retina epigenetics

## Abstract

The study of epigenetics has explained some of the ‘missing heritability’ of age-related macular degeneration (AMD). The epigenome also provides a substantial contribution to the organisation of the functional retina. There is emerging evidence of specific epigenetic mechanisms associated with AMD. This ‘AMD epigenome’ may offer the chance to develop novel AMD treatments.

## Introduction

Advanced age-related macular degeneration (AMD) manifests as either choroidal neovascularization (wet AMD) or geographic atrophy (late dry AMD). Drusen, a mixture of lipid and protein components, is the earliest histopathological and clinical finding observed in the early stages [1, 2].

Genetically confirmed monozygotic twins can present initially with similar early macular pathology. However, in the late stages of the disease, phenotypes can differ in terms of the time of onset and type of late disease (wet or dry) or indeed one twin may develop AMD and the other does not [3].

Allele-specific methylation patterns can differ in regions surrounding AMD candidate genes between monozygotic twins. Therefore, epigenetic mechanisms may explain some of the phenotypic variability in AMD which cannot be accounted for by Mendelian genetics [4].

### Epigenetics in evolution and in human disease

Forty years ago, it was pointed out that natural selection does not randomly result in phenotypic variations and that organisms interact with the environment to produce direct evolutionary change [5]. This notion was based on an earlier discovery by Waddington that acquired characteristics are inherited in response to environmental stimuli [6]. Denis Noble reiterated Waddington’s theory by explaining that, in the absence of new mutations, acquired characteristics can be inherited and integrated into the genome [7].

The Mendelian laws of dominance, segregation, and independent assortment seem inadequate to explain common chronic diseases with adult onset such as coronary heart disease, hypertension, diabetes or cancer and others in the context of the genetics of complex human disease. Diseases influenced by multiple genetic and environmental factors, and the ability to identify hundreds of thousands of common variants, through Genome Wide Association Studies (GWAS), some of which may predispose to such diseases, have recently changed the concept of inheritance for polygenic traits.

Both from an evolutionary as well as from a disease pathogenesis point of view, the so called neo-Darwinian and Mendelian principles that attributed both evolution and disease inheritance to the ‘absolute power of genes’ were seriously challenged by the introduction of a new concept that we now call ‘epigenetics’, a term based on the Greek word ‘epi’ as one of the compound words that means’ above’, therefore ‘above genetics’.

An epigenetic modification means chromosomal modification in the absence of a DNA sequence change. Depending on classification systems, chromatin alterations can be considered either as heritable (Operational definition of Epigenetics) or non-heritable (Epigenomics Mapping Consortium) [8, 9].

DNA methylation, histone modification, chromatin remodelling and non-coding RNA-mediated gene silencing, are all mechanisms determining the epigenetic landscape and gene expression responsible for cellular memory [10].

It is the epigenome that contributes substantially to the organisation of a functional retina, through fine-tuning gene expression patterns. This leads to the generation of great diversity in different cell types and in the molecular machinery, in both the developing and the ageing retina [11]. Establishing control of gene expression, epigenetic signals have been found to be related to retinal development and moreover to retinal disease, and specific epigenetic changes to be associated with the most devastating complex eye disease in the elderly population: AMD [12].

In the last 10 years, high-resolution studies of epigenetic states at the individual cellular level have become possible. This is because of high-throughput sequencing technology and whole transcriptome sequencing (RNA-seq) as well as developments in DNA barcoding and microfluidic technologies [13].

### AMD pathogenic mechanisms and interaction of genetic/environmental risk factors

Accumulation of extracellular deposits of lipid, cellular debris and proteins, known as drusen, has long been established as the earliest hallmark of AMD pathogenesis. Despite the fact that there is currently no single animal model that develops all signs of AMD [14], simulating long-term oxidative stress and inflammatory conditions has been commonly used to facilitate AMD model development of oxidative damage. Neovascularization is thought to be a response to hypoxia caused by drusen deposits interrupting the flow of oxygen from the choroid through the RPE and into the retinal cells [15]. The role of inflammation in developing neovascular AMD is poorly understood. For example, IL-18, a pro-inflammatory cytokine, inhibits secretion of VEGF which lowers VEGFR-2 expression [16, 17]. However, low grade chronic inflammation is thought to cause AMD progression. It has also been shown that modulation of the immune response in the ageing retina aids normal tissue function [18].

Amyloid beta (Ab) is a main component of drusen [19, 20], and Ab1-40 is thought to be the principal form found in drusen [21]. Activation of the complement system follows elevation of Ab deposits and leads to the induction of upregulated angiogenic factors [22, 23]. Transgenic mouse models overexpressing Ab develop RPE degeneration and basal deposits, similar to dry AMD patients [24, 25]. The gene expression profile in response to Ab1-40 stimulation in human RPE cells has been reported as well [26, 27]. Elevated Ab deposits induce a pro-inflammatory microenvironment and subsequently activate the complement system [28, 29].

NLRP3 inflammasome activation has been implicated in the pathogenesis of geographic atrophy, induced by anti-infectious components in drusen. These components include complement components, apolipoprotein E, amyloid Aβ proteins, vitronectin, immunoglobulins and C1Q [13]. This leads to caspase-mediated processing of the cytokines, interleukin IL-1b and IL-18, which in turn mediates innate and adaptive immunity. Thus, indicating that ocular infection may have a role in AMD pathogenesis [30-35]. Wen et al. suggested an explanation for the varying shapes and sizes in drusen. This was that microorganisms/bacteria play a role in drusen formation. They speculated that antimicrobial responses in drusen can be significantly affected by genetic defects in *CFH* and *HTRA1*. They reached their conclusions after subretinal injection of the bacterium *Bacillus megaterium* in non-human primate models that induced drusen-like pathology [13].

The functional effects of the non-synonymous *CFH Y402H* polymorphism have been shown before: Klein et al. found a polymorphism in exon 9 of *CFH* (rs1061170) that represents a tyrosine-histidine change at amino acid 402 in a region of CFH that binds heparin and C-reactive protein. The variant increases the risk for AMD 4.6-fold in individuals heterozygous for the haplotype and 7.4-fold in individuals homozygous as it appears to reduce the ability of CRP to inhibit AP complement activation [36]. *C3* has been shown to result in reduced binding to CFH and reduced complement regulation leading to increased membrane attack complex (MAC) deposition at the choriocapillaris [37, 38]. The association of AMD pathogenesis with chromosome 10q26, which surrounds *ARMS2* and *HTRA1*, has also been demonstrated in many studies although the debate is ongoing as to the causal allele and underlying pathogenic mechanism [39].

Gene–environment interaction has been shown to affect retinal cells in which gene expression changes in response to endogenous or exogenous stimuli [40]. Genetic and environmental risk factors (smoking, light exposure, nutrients), as well as age- and race-related predisposing factors may lead to oxidative damage and inflammation Thus causing early AMD to progress into the advanced stages of geographic atrophy and choroidal neovascularization [41]. AMD patients of western European descent suffer the dry form of AMD (geographic atrophy), the strongest genetic risk factor being CFH Y402H, whereas Asian AMD patients are more likely to suffer wet AMD or polypoidal choroidal vasculopathy (PCV) associated with genetic polymorphism in the HTRA1 gene [42]. Phenotypic variation suggests environmental factors, not just different gene combinations, may have a significant input in AMD development.

## DNA methylation/demethylation patterns and histone modifications in the development of AMD

It is now known that what is perceived as ‘mobile DNA’ or to use the most appropriate term ‘transposable elements’ (TEs) contribute to normal biological processes. This is very different than the role initially attributed to them as simply ‘genetic parasites’ that might be related to human disease [43, 44]. It is also now known that TEs contribute to the evolution of non-coding RNAs, and that they can copy themselves in the genome so that they will form new regulatory elements which contribute to the ‘rewiring’ of gene regulatory networks [45-49] Recruitment of transcription factors heavily depends on the incorporation of *cis*-regulatory elements by TEs [47].

During early development, DNA is demethylated and TEs are released, replicated and enter the germ line [50-52]. The most important suppressive mechanism in somatic tissues that silences TEs is DNA methylation [53]. Therefore genome integrity relies on a balance between the benefits and the negative effects of TEs regulated by the epigenetic system and mainly by the methylation/demethylation DNA status. Genome-wide hypomethylation can lead to chromosome instability and increased mutation rates [54].

The modification of DNA by the addition of a methyl group to cytosine changes the electrostatic nature of chromatin and can be the result of damaging proteins, lipids and free radicals [55, 56]. The addition of a methyl group to the C5 position of cytosine bases, that are followed by guanosine bases within the DNA sequence (5′-CpG-3′), is associated with the repression of gene expression. Unmethylated CpG islands are a feature of actively expressed genes and unexpressed genes generally have methylated CpG islands near their transcription start sites [11].

Writers can add, readers can interpret and erasers can remove epigenetic modifications on chromatin. Histone acetyltransferases add, and histone deacetylases (HDAC) remove acetyl groups to and from histone lysine residues, respectively [11]. HDAC inhibitors have been utilised in clinical trials to treat cancer and could be used for the treatment of retinal degenerative diseases [57].One class of HDACs known as sirtuins has been implicated in the ageing process [58].

**Fig. 1:**
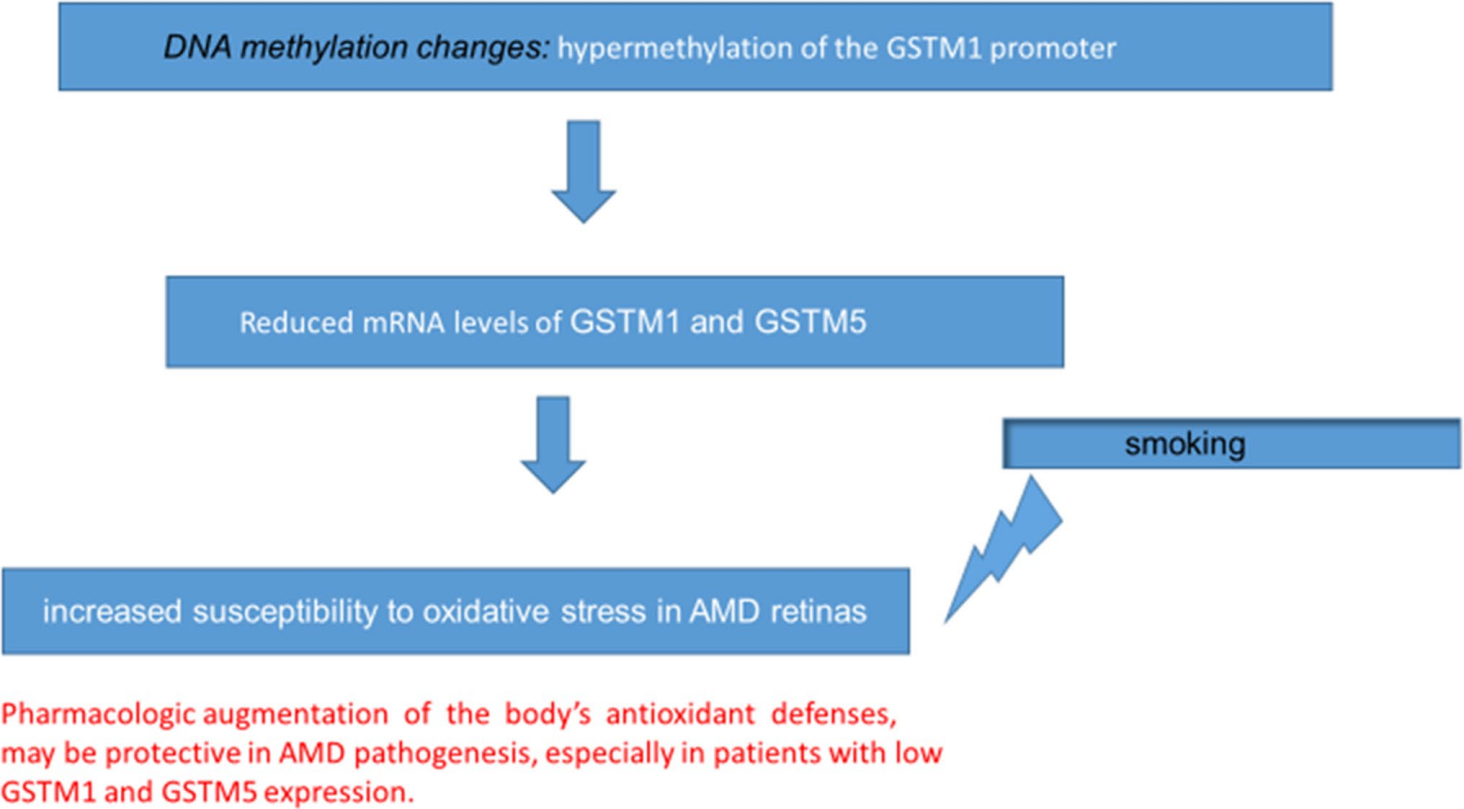
DNA methylation is associated with altered gene expression in AMD [59]

What has been already noted before, regarding the effect of DNA methylation changes and antioxidant gene expression in AMD, is that mRNA levels of glutathione *S*-transferase isoforms mu1 (*GSTM1*) and mu5 (*GSTM5*) were significantly reduced in AMD patients vs age-matched controls in the retinal pigment epithelium (RPE)/choroid and neurosensory retina. This suggests that *GSTM1* and *GSTM5* undergo epigenetic repression in AMD RPE/choroid. This may increase susceptibility to oxidative stress in the retina of AMD patients (Fig. 1) [59].

A significantly decreased level of methylation of the *IL17RC* promoter in AMD patients has been reported. The interleukin 17 receptor C (*IL17RC*) gene serves as an essential subunit of the IL-17 receptor complex that mediates the signal transduction and pro-inflammatory activities of *IL-17A* and *IL-17F* [60].

**Fig. 2:**
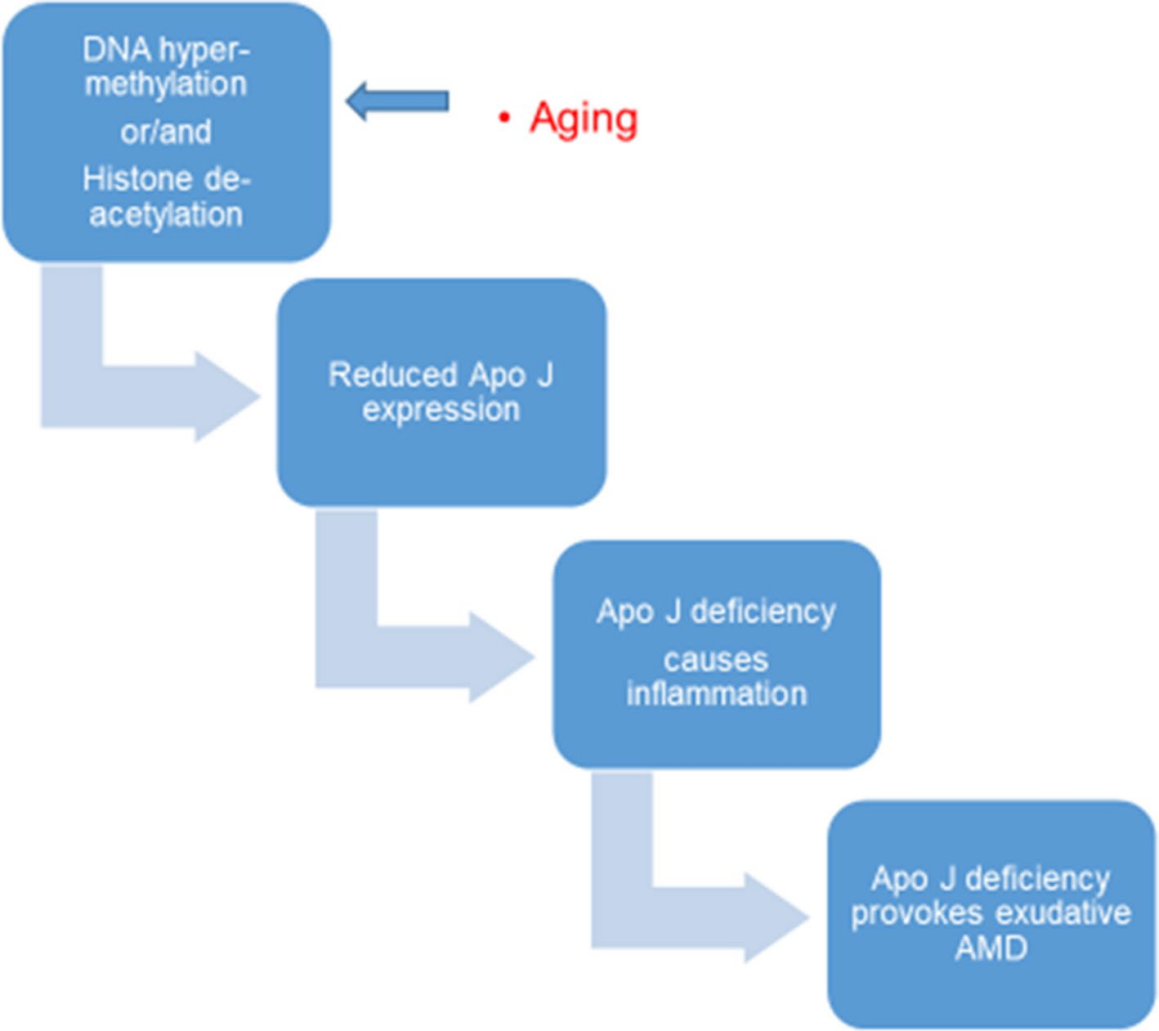
DNA methylation and histone acetylation status may affect AMD pathogenesis via the inhibition of angiogenesis and inflammation [64, 65]

DNA methylation is related to histone acetylation status and both DNA methyltransferase (DNMT) and histone deacetylase (HDAC) inhibitors inhibit angiogenesis causing histone hyperacetylation and selective gene transcription. This is valuable knowledge when exploring candidate cancer treatments [61-63]. Ageing affects both DNA methylation and histone acetylation status through the clusterin/apolipoprotein J (apo J) and vitronectin complement regulatory proteins. These bind to the membrane, attack complexes and prevent cytolysis. Inflammation and development of neovascular AMD may be epigenetically regulated as they are aggravated by the deficiency of the complement regulatory proteins above, the expression of which is related to the promoter of clusterin containing a CpG-rich methylation domain. Expression (and secretion) levels of clusterin mRNA and protein in ARPE-19 cells increase when treated with DNMT and HDAC inhibitors (Fig. 2) [64, 65].

Oliver et al. investigated DNA methylation levels in peripheral blood samples and frozen sucrose gradient-treated peripheral retinas of AMD patients with either geographic atrophy or neovascularization compared with unaffected control patients [66]. In the only genome-wide epigenetic study of AMD to date, they observed hypomethylation at the *ARMS2/HTRA1* locus and hypermethylation at the *protease serine 50 (PRSS50)* locus in AMD patients compared with controls. The *ARMS2/HTRA1* locus is one of the strongest loci genetically associated with AMD. The finding that hypomethylation at the *ARMS2/HTRA1* locus associates with AMD, supports a role for either or both of these genes being involved in the development of disease. The *PRSS50* locus had not previously been associated with AMD risk. It is not clear whether DNA methylation patterns of easily accessible tissues like whole blood truly represent the epigenetic phenotype in inaccessible tissues such as retina. Environmental factors such as smoking have an impact on multiple tissues and therefore epigenetic alterations related to certain environmental effects may indeed follow similar patterns in different tissues. Interestingly, methylation differences in the *PRSS50* promoter were comparable in both blood and the retina.

A question arising, when studying epigenetic regulation in post mortem tissue, is the degree that tissue processing procedures may affect detection of DNA methylation levels. Such studies are common practise in the investigation of epigenetic changes in the human retina of AMD patients. This is relevant to the resistance to degradation and therefore the quality of the DNA molecule. This has been investigated by Rhein et al. who analysed blood methylation levels, compared to brain methylation values, and observed significant variation of DNA quality in different tissues [67]. This should be considered when interpreting data on epigenetic phenomena, and especially when investigating blood and retinal tissue of AMD patients, where low levels of epigenetic differences can have a substantial impact on data interpretation.

One class of HDACs, known as sirtuins, has been implicated in the pathogenesis of the metabolic syndrome, neurodegenerative diseases, the inflammatory response, circulatory system diseases, neoplasms and other age-related diseases [68]. Processes such as gene expression, cellular metabolism, DNA repair, apoptosis, development, inflammatory response and neuroprotection have been linked to modulation of sirtuins [69, 70].

Chronological age has been shown to have a significant effect on methylation levels and DNA methylation-based biological markers of ageing or ‘epigenetic clocks’ have been proposed [71, 72]. Greater methylation age refers to accelerated epigenetic ageing and has been linked to age -related frailty [73], lung cancer [74] and Parkinson’s disease [75], as well as all-cause mortality [76, 77]. It does not necessarily correlate with chronological age.

Stevenson et al. hypothesised that a faster running epigenetic clock would associate with greater levels of systemic inflammatory biomarkers. To study this, they used the Lothian Birth Cohort 1936. They phenotyped participants of 4 waves up to now at mean ages of 70, 73, 76 and 79 with a range of health outcomes, lifestyle factors, psycho-social variables, genetics, epigenetics and cognition. They demonstrated positive associations between all inflammatory biomarkers tested and extrinsic epigenetic age acceleration, suggesting a faster running epigenetic clock is associated with an increased inflammatory profile [78].

## Chromatin remodelling and accessibility in AMD

To fully understand the meaning of recent landmark studies in AMD epigenetics one needs to be familiar with an epigenetics related term called ‘chromatin accessibility’. Accessible chromatin is the physical contact of nuclear macromolecules to chromatin DNA and it is partly determined by chromatin binding factors that prevent DNA access. Chromatin accessibility is regulated by the binding of transcription factors that changes through modulation of the nucleosome which is the core structural element of chromatin. Accessibility ranges from closed to highly accessible chromatin, which reflects a dynamic state of regulatory capacity at a genetic locus [79]. Histone deacetylation and methylated chromatin contribute to ‘closed’ nucleosome formation (heterochromatin) that inhibits DNA transcription whereas the ‘open’ chromatin structure (euchromatin) allows transcription via binding of transcription factors to DNA [80, 81]. Stability of constitutive heterochromatin is essential to maintain genome integrity. Therefore, imbalance in the ratio of herochromatin:euchromatin, due to certain environmental stimuli, could lead to diseases such as cancer and ocular disorders including AMD [82].

Wang et al. demonstrated that global decrease in chromatin accessibility occurs in the RPE, even in early dry AMD. This suggests that dysfunction in the RPE drives disease onset. Epigenetic changes in RPE cells might be a critical factor regulating the early stages of the disease [83]. Interestingly, cigarette smoke treatment of induced pluripotent stem cell (iPSC) -derived RPE could trigger changes in chromatin accessibility. This resulted in a global decrease in chromatin accessibility after treatment, similar to changes in RPE tissue from AMD samples. Additionally, this study showed that histone deacetylase genes and specifically *HDAC11* overexpression may be in part the cause for the global decrease of chromatin accessibility associated with AMD progression.

The above study provides new insight of the role that epigenetics may be playing in AMD pathogenesis since local AMD-associated variants had no impact on the observed differences in chromatin accessibility. Decreased chromatin accessibility and differences in nuclear permeability, which are regulatory mechanisms of the epigenome, seemed to supersede the importance of known AMD risk genetic variants.

## Micro-RNAs and post-transcriptional modulation in AMD

MicroRNAs (mi-RNAs) are single-stranded short (21–23 nucleotides long) non-coding RNAs that are involved in post-transcriptional modulation [84]. Translational repression and mRNA degradation are the outcomes of base pairing between miRNA and mRNA [85]. miRNA-mRNA affinity is affected mainly by single nucleotide polymorphisms (SNPs) and may have an impact on transcription of target genes [86]. miRNAs bind to one or more partially complementary sites in the target mRNA in the 3′ untranslated region (3′ UTR) and recruit a large protein complex known as the RNA-induced silencing complex to target mRNAs which are subsequently silenced [87].

Endothelial cell proliferation, migration and actin cytoskeleton organization were affected by miR-21 overexpression. This reduced angiogenesis in a model of pathologic CNV [88].

Shen et al.’s microarray analysis of miRNAs in ischaemic retina confirmed downregulation of three miRNAs: miR-31, miR-150 and miR-184 and candidate target genes were identified for each one of the three: *Pdgfb* and *Hif1a* for miR-31, *Vegf *and *Pdgfb* for miR-150 and *Frizzled4* for miR-184 [89]. Injection of premiR-31 reduced VEGF in ischaemic retina indirectly, possibly through reduction of HIF-1a. This paper showed that simultaneous alteration of multiple gene products by mi-RNAs modulated control of angiogenesis. This may have direct relevance to neovascular AMD.

Given the importance of hypoxia and ROS production in RPE cell damage, investigating the interaction between miR-23a and apoptotic factors such as Fas (apoptosis antigen 1) in oxidative stress seemed a reasonable approach [90]. Lin et al. showed that miR23 expression was downregulated in the RPE of AMD patients whereas miR23 overexpression reduced RPE cell death through downregulation of Fas, proving an anti-apoptotic effect of miR-23a that protected RPE cells.

Treatment of human RPE cells with inflammatory cytokines IFN-g, TNF-a and IL-1b increases miR-155 expression. This controls the response of RPE cells to chronic inflammation which is important in AMD pathogenesis [91].

Upregulated brain- and retinal-abundant miRNAs, including miRNA-9, miRNA-125b, miRNA146a and miRNA-155 have been shown to lead to CFH deficiency. This may drive inflammatory neurodegeneration in both Alzheimer’s disease and AMD. Interestingly, these diseases share several common pathological features [92, 93].

We know Alu transcripts, the most common form of transposable elements, are potentially disruptive and can result in inherited disorders. The miRNA-processing enzyme DICER1 is reduced in the RPE of humans with GA and that miRNA dysregulation promoting GA cannot be excluded [94]. DICER1 is inhibited by Alu RNA antisense oligonucleotides and induces RPE cytotoxicity. The exact mechanism of cytotoxicity induced by Alu-RNA transcripts is still largely unknown. However, it has been suggested that exposure to Alu RNA, or Dicer1 deficiency activates the NLRP3 inflammasome and triggers toll-like receptor (TLR) independent myeloid differentiation primary response 88 adapter protein (MyD88) signalling via IL-18 in the RPE. This represents a pathway leading to GA [95].

Haematoxylin and eosin-stained sections of retinal pigment epithelium (RPE)/neural retina tissue of C57BL/6 mice intravitreally injected with Ab1-40 or PBS showed degenerative changes, and immunofluorescence testing demonstrated apoptosis within the retina after Ab1-40 injection [96]. In the same study, miRNA expression profiling proved differential expression of miRNAs between the Ab model and the control group. The investigators used multiple miRNA databases to identify potential miRNA genes. They correlated these with molecular pathways (functional analysis) and calculated p values to determine statistical significance. Functional analysis of the 61 differentially expressed miRNAs identified the top 10 most relevant pathways and the targeted genes of the regulated miRNAs. miR-155 shown to lead to CFH deficiency before, was shown to be upregulated by Ab in this study, and oxidative stress already found to be crucial in dry AMD pathogenesis was shown to activate pathways such as the mitogen-activated protein kinase (MAPK) signalling pathway already known for its implication in AMD pathogenesis [97], through miRNA differential expression.

Kim et al. demonstrated that miR-33 regulates ATP-binding cassette transporter A1 (*ABCA1*) which regulates ApoE lipidation, and this in turn has an effect on Ab levels in the brain and showed the role that dysregulation in the metabolism of Ab may have in the pathogenesis of Alzheimer's disease which may be similar to the role that Ab-induced upregulation of miR-33 in the retina/RPE plays in AMD pathogenesis [98].

Popp et al. noted that a small number of SNPs in the 3′ untranslated region (3′UTR) have been associated with AMD. They therefore tested allele-specific dysregulation of miRNA binding ability in the 3′UTR of *IL-17A* in AMD patients [99]. SNP rs7747909 showed the strongest association, even after correction for age, gender and smoking status although this was not replicated in a second cohort of Caucasian AREDS patients. This SNP can modulate hsa-miR-4480 binding and therefore the in vitro degradation of *IL-17A* mRNA*.*

## Epigenetic mechanisms and treatment of AMD

As discussed earlier, DNA methylation/demethylation and histone acetylation/deacetylation patterns are thought to be the main epigenetic regulatory mechanisms affecting the critical balance between the benefits and the negative impact of TEs. He et al. showed the complexity of such epigenetic regulation of TEs in embryonic stem cells. The authors suggested that the regulation of TEs is a function of overlapping epigenetic pathways, and that a major task of the epigenetic system is to manage the expression of TEs [100].

To fully explain complex epigenetic pathways is challenging due to epigenetic patterns depending on type of tissue, age and state of a disease [101, 102].

However, there are efforts to utilise knowledge of epigenetic modifications associated with retinal disease to identify potential therapeutic targets. The aim would be to fix the aberrant chromatin modifications that cause expression of genes of relevance to the development of AMD [11].

### DNMT and HDAC inhibitors

DNMT inhibitors (DNMTIs) and HDAC inhibitors (HDACIs), aiming to block methylation and histone deacetylation have been suggested as potential AMD treatments. These may act via enhancing complement inhibition in the early AMD stages, as a result of an increase in the expression levels of clusterin mRNA and its protein expression, or via inhibition of angiogenesis in late neovascular AMD [64, 65, 103].

5-Aza-2 0-deoxycytidine (AZA), a DNMTI, upregulates clusterin expression in RPE cells via hypomethylation of the CpG islands in its promoter region. This epigenetic mechanism appears to inhibit angiogenesis. Therefore, this drug may be useful in inhibiting neovascularization in late AMD [64]. DNMT inhibitors also inhibit tumour angiogenesis and decrease tumour volumes in brain endothelioma cells in mice [103].

Trichostatin A (TSA), a HDACI, supresses expression of retinal tumour necrosis factor-a (*TNF-a*) attributing to TSA a protective role against ischaemia and against the activity of matrix metalloproteinases (MMPs) associated with *TNF-a* receptor stimulation [104]. Valproic acid, another HDACI, reduces neovascularization as well as drusen formation [64, 65, 105]. Sirtinol, an inhibitor of sirtuins, reverses such changes and reduces proliferation of choroidal endothelial cells [106]. Nicotinamide, another sirtuin inhibitor, was shown to reduce secretion of pro-angiogenic factors in RPE cells. It similarly appears to be a potential treatment to inhibit angiogenesis in late AMD [107]. Additionally, investigators showed that genes related to angiogenesis, angiopoietin 2 and endothelial cell nitric oxide synthase were downregulated by HDACIs [108, 109].

Tanito et al. tested sulforaphane (SF), a natural compound in broccoli sprouts, as a potential inducer of Thioredoxin (Trx) that protects retinal and RPE cells from light-induced damage. They found that SF indeed induced expression of redox proteins via inhibition of HDACs, when cells were exposed to the inflammatory stimulus, through modulation of the expression of the *TRx* gene.[110].

### Alternative chromatin structure modulators

Normal RPE cells produce extracellular matrix (ECM) proteins and angiogenic factors. Uchida et al. investigated the effect of all-trans-retinoic acid on the production of an ECM protein, thrombospondin-1 (TSP-1), pigment epithelium-derived factor (PEDF) and vascular endothelial growth factor (VEGF) by RPE cells and found that retinoic acid a product of vitamin A metabolism increased the release of TSP-1 and PEDF from human RPE cells whereas expression of TSP-1 and PEDF from the RPE layer of vitamin A-deficient mice was significantly decreased. Thus, they suggested that vitamin A deficiency upregulates the expression of ECM proteins, TSP-1 and PEDF and therefore vitamin A modulates the expression of angiogenesis-related genes via altering the structure of chromatin [111]. 9-*cis* Retinoic acid increased the rate of apoptosis in foetal bovine aortic endothelial cells and it was therefore shown that 9-*cis* retinoic acid mediates vascular endothelial apoptosis whereas confluent foetal bovine aortic endothelial cells cultured with a confluent layer of RPE reduced the 9-*cis* retinoic acid-induced apoptosis rate [112]. Loss of tight and adherens junctions’ integrity in the RPE can disrupt photoreceptor homeostasis [113-117] and degradation of anti-apoptotic conditions that inactivate pro-apoptotic and pro-inflammatory signalling pathways make RPE cells vulnerable to normal pro-apoptotic stressors. This is what Bhattacharya et al. suggested [118]. This links disruption of the p53/Mdm2 apoptotic pathway with age-related increased susceptibility of RPE cells to apoptosis. Therefore, anti-apoptotic proteins, such as B-cell CLL/lymphoma 2 (Bcl-2) and resveratrol a polyphenol found in red wine that acts as a sirtuin activator and inhibits caspase 3 activation could restore the integrity of the RPE and prevent apoptosis [119].

### mRNA targeting: a potential therapeutic approach

The therapeutic potential of miR-21 in pathologic angiogenesis was shown by a significant reduction in blood vessel density after intravitreal injection of premiR-21 in a laser-induced murine model of CNV [120]. Intravitreal injection of premiR-31 into the eyes of mice with ischemic retinopathy caused significant reductions in platelet-derived growth factor-B (PDGF-B) and HIF-1a and a similarly marked decrease of VEGF and/or PDGF-B levels was noted after injection of premiR-31 or premiR-150. They also had an anti-angiogenic effect in ischemic retina [89, 121, 122]. Simultaneous alteration of several gene products in the angiogenesis cascade by miRNAs may be an attractive treatment option for neovascular AMD. Theoretically this would produce a prolonged clinical response. This is currently missing from conventional anti-angiogenic treatments of the disease.

Lin et al. showed that overexpression of miR-23a reduced cell death through binding to apoptotic factors (Fas) involved in ROS-mediated cell death, and therefore they proved miR-23a is cytoprotective towards RPE cells by prevention of apoptosis [90].

miR-155 has the potential to modulate the response of the RPE cells to inflammatory stimuli and to serve as a target for therapeutic intervention in AMD [123-126].

Upregulation of brain- and retinal-abundant miRNAs, including miRNA-9, miRNA-125b, miRNA146a and miRNA-155 implicated in the pathogenetic mechanism of CFH deficiency was shown by Lukiw et al. Thus, anti-miRNA therapeutics seem plausible for the management of inflammatory neurodegeneration in diseases like Alzheimer’s and AMD [92].

Alu RNA antisense oligonucleotides inhibit DICER1 depletion-induced RPE cytotoxicity and therefore, Alu RNA inhibition or DICER1 augmentation may serve as potential therapeutic options for geographic atrophy [94]. Whether this means Alu RNA inhibition will prevent atrophy or will facilitate tissue re-growth or both remains to be further investigated.

## Future research

The only randomised clinical trial that looked at a potential epigenetic therapeutic intervention was the Women’s Antioxidant and Folic Acid Cardiovascular Study (WAFACS) that evaluated whether combined treatment with folic acid, vitamin B_6_ and vitamin B_12_ could prevent cardiovascular events among women at high risk. The incidence of AMD was examined as well. The investigators suggested that daily supplementation with folic acid/B_6_/B_12_ may reduce the risk of AMD partly due to homocysteine lowering levels or due to the antioxidant effect of folic acid and B vitamins, and enhancement of endothelial nitric oxide levels in the choroidal vasculature [127].

**Fig. 3:**
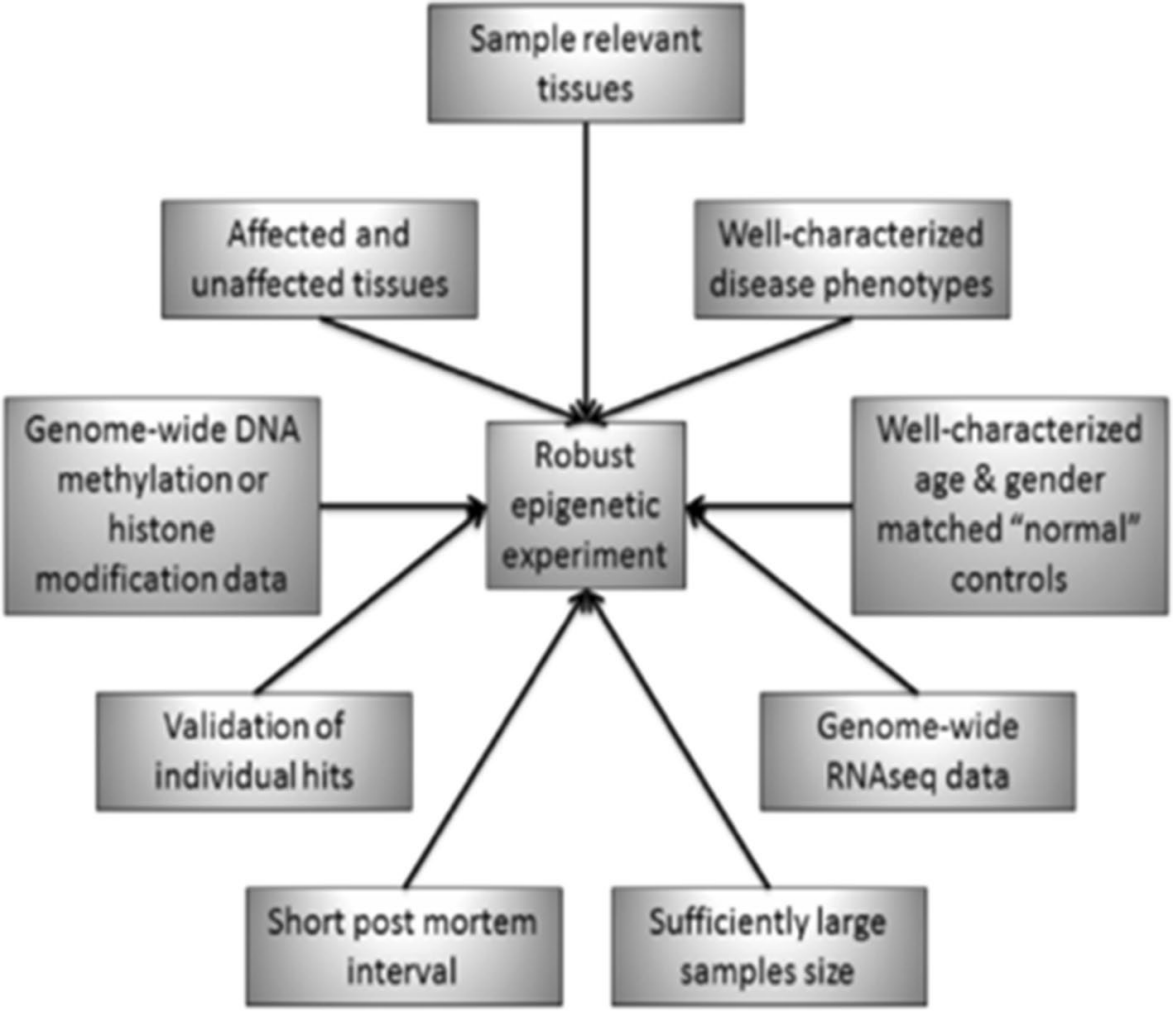
Ideal experimental setup for genome-wide epigenetic experiments as displayed by Pennington and DeAngelis [11]

Pennington et al. summarised the conditions that would be contributing to the ‘ideal experimental setup for genome-wide epigenetic experiments so that useful epigenetic data are produced. They explained, genes differentially methylated in specific disease states could be identified this way, closely associated with the causative mechanisms that lead to disease development and progression [11] (Fig. 3). AMD phenotypic variation from early to advanced stages may be further elucidated on a similar basis and therapeutic targets then set.

The Human Epigenome Project [128] encompasses high-resolution genome-wide epigenetic profiles and high-throughput screening procedures. It offers the chance to develop the epigenetic landscape of the ‘AMD epigenome’. Similar to results from studying the cancer epigenome, future—epigenetics-based—treatments may result. This could transform the future of AMD treatment. More effective and earlier treatments for AMD are needed and so this approach is worth exploring further.
